# Immunomodulatory intervention in sepsis by multidrug-resistant *Pseudomonas aeruginosa *with thalidomide: an experimental study

**DOI:** 10.1186/1471-2334-5-51

**Published:** 2005-06-26

**Authors:** Evangelos J Giamarellos-Bourboulis, Nikolaos Bolanos, George Laoutaris, Vassilios Papadakis, Vassilios Koussoulas, Despina Perrea, Panayotis E Karayannacos, Helen Giamarellou

**Affiliations:** 1 4th Department of Internal Medicine, University of Athens, Medical School, Athens, Greece; 2 Laboratory of Experimental Surgery and Surgical Research, University of Athens, Medical School, Athens, Greece; 3 Center for Biomedical Research, Academy of Athens, Athens, Greece

## Abstract

**Background:**

Thalidomide is an inhibitor of tumour necrosis factor-alpha (TNFα) that has been proven effective for the treatment of experimental sepsis by *Escherichia coli*. It was tested whether it might behave as an effective immunomodulator in experimental sepsis by multidrug-resistant (MDR) *Pseudomonas aeruginosa*.

**Methods:**

Sepsis was induced by the intraperitoneal injection of 1 × 10^8 ^cfu/kg inoculum of the test isolate in a total of 109 Wistar rats divided in three groups as follows: group A controls; group B administered seed oil 30 minutes before bacterial challenge; and group C administered 50 mg/kg of thalidomide diluted in seed oil 30 minutes before bacterial challenge. Blood was sampled for estimation of endotoxins (LPS), TNFα, interferon-gamma (IFNγ), nitric oxide (NO) and malondialdehyde (MDA). LPS was measured by the QCL-1000 LAL assay, TNFα and IFNγ by ELISA, NO by a colorimetric assay and MDA by the thiobarbiturate assay.

**Results:**

Mean (± SE) survival of groups A, B and C were 18.60 ± 1.84, 12.60 ± 0.60 and 30.50 ± 6.62 hours (p of comparisons A to C equal to 0.043 and B to C equal to 0.002). Decreased TNFα and NO levels were found in sera of animals of group C compared to group A. Plasma levels of LPS, MDA and IFNγ did not differ between groups.

**Conclusion:**

Intake of thalidomide considerably prolonged survival in experimental sepsis by MDR *P.aeruginosa *an effect probably attributed to decrease of serum TNFα.

## Background

Nosocomial infections are commonly caused by multidrug-resistant Gram-negative pathogens. Management of these infections is difficult due to the lack of potent antimicrobial agents; thus a target for immunomodulatory intervention is created [1]. Thalidomide is an old regimen that has been proved potent in reducing the half-life of mRNA of the gene of tumour necrosis factor-alpha (TNFα) in human monocytes [2]. Its anti-angiogenic and anti-TNFα properties have led to its application for the treatment of erythema nodosum leprosum, of cutaneous lupus erythematosus, of Behçet's syndrome, of multiple myeloma and of HIV-related aphthous ulcers and wasting syndrome [3,4].

In a model of experimental sepsis by *Escherichia coli*, thalidomide was proved very effective in reducing serum levels of TNFα, a phenomenon that was associated with refraining of evolution to sepsis [5]. However, its effect on survival was not assessed. The immunomodulatory benefit of thalidomide would be of considerable importance for sepsis induced by multidrug-resistant isolates. The present study was designed to evaluate thalidomide in experimental sepsis by multidrug-resistant *Pseudomonas aeruginosa*. Interest was focused on the effect of thalidomide on a) survival after bacterial challenge, and b) serum levels of pro-inflammatory mediators.

## Methods

### Animals

A total of 109 male Wistar rats were enrolled in the study. Their mean (± SD) weight were 257.2 ± 40.2 g. The study received permit from the Veterinary Directorate of the Perfecture of Athens according to the Greek legislation in conformance to the Council Directive of the European Community. Rats were housed in metal cages and had access to tap water and standard balanced chow *ad libitum*. Temperature ranged between 18 and 22°C, relative humidity between 55 and 65% and the light/dark cycle was 6 am/6 pm.

### Bacterial isolate

One multidrug-resistant blood isolate of *P. aeruginosa *derived from a patient with nosocomial sepsis was applied. Minimal inhibitory concentrations (MICs) of ticarcillin/clavulanate, piperacillin, ceftazidime, imipenem, meropenem, ciprofloxacin and amikacin were determined by a microdilution technique of a 0.1 ml final volume. MIC was considered as the lowest concentrations of the tested antimicrobial limiting visible bacterial growth after 18 hours of incubation at 35°C. The isolate was stored as multiple aliquots in skim milk (Oxoid Ltd, London, UK) under -70°C. One aliquot was removed from the fridge before each experiment. Single colonies were incubated at 37°C in 10 ml of Mueller-Hinton broth (Oxoid Ltd) for eight hours to yield a log-phase inoculum that was applied for bacterial challenge.

### Study design

Animals were divided into three groups of treatment, as follows:

• Group A (n = 40), controls; in 20 of these animals survival was recorded after bacterial challenge and 20 were sacrificed five hours after bacterial challenge.

• Group B (n = 20), animals pre-treated with linseed oil 30 minutes before bacterial challenge; survival was then recorded.

• Group C (n = 49), animals pre-treated with thalidomide 30 minutes before bacterial challenge; survival was recorded for 24 and another 25 were sacrificed five hours after bacterial challenge.

Thalidomide was supplied as a white amorphous powder (ICN Biomedicals GmbH, Thüringer, Germany) insoluble in water. It was diluted in commercial linseed oil and it was administered via a gastric tube in animals of group C at a dose of 50 mg/kg, based on previous results [5]. The respective dose of linseed oil administered was 2 ml/kg. The same dose was administered in animals of group B. A 1 × 10^8 ^cfu/kg inoculum of the test isolate was injected intraperitoneally in all animals.

Survival was recorded at 12-hour time intervals. Five hours after bacterial challenge, a midline abdominal incision was performed. Intestines were displaced to the left and the inferior vena cava was recognized and punctured with a 19-gauge needle. Ten ml of blood was collected into pyrogen-free syringes and applied for culture and for the estimation of endotoxins (LPS), of tumour necrosis factor-alpha (TNFα), of interferon-gamma (IFNγ), of nitric oxide (NO) and of malondialdehyde (MDA). Animals were then sacrificed by an intramuscular injection of pentothal. The 5-hour time interval was selected as the most appropriate for blood sampling after estimation of pro-inflammatory mediators in groups of animals inoculated by the test pathogen with hourly time differences between each sampling (data not shown).

### Blood culture and estimation of LPS, cytokines, NO and MDA

One ml of blood was added into flasks with growth medium (Becton Dickinson, Cockeysville, Md) and incubated at 35°C for a total of seven days. Identification of Gram-negative isolates from blood cultures was made by the API20NE test (bioMérieux, Paris, France).

Sampled blood was added into pyrogen-free tubes (Vacutainer, Becton Dickinson), and centrifuged; serum was kept refrigerated as multiple aliquots at -70°C until assayed. For the determination of LPS, serum was diluted 1:10 with pyrogen-free water (BioWhitaler, Maryland, USA) and incubated for five minutes at 70°C. LPS were then measured by the QCL-1000 LAL assay (BioWhitaker, Maryland, USA, lower detection limit 1 EU/ml). TNFα and IFNγ were estimated in serum by an enzymoimmunoassay (Diaclone, Paris, France). Lower detection limits for TNFα and IFNγ were 10 and 10 pg/ml respectively. All determinations were performed in duplicate.

Nitric oxide (NO) was estimated in serum samples by a colorimetric assay based on the production of velvet color after sequential addition of NADH and nitrate reductase (Assay Designs Inc., Ann Arbor Minnesota, USA). Optical density was read at 570 nm (Hitachi Spectrophotometer).

Lipid peroxidation in serum was assessed by the estimation of the concentration of malondialdehyde according to the thiobarbiturate assay, as already described [6,7]. Briefly, a 0.1 ml aliquot of each sample was mixed to 0.9 ml of trichloroacetic acid 20% (Merck, Darmstadt, Germany) and centrifuged at 12,000 g and 4°C for 10 minutes. The supernatant was incubated with 1 ml of PBS (pH: 7.2) and 1 ml of thiobarbituric acid 0.6% (Merck) for 20 minutes at 90°C. Optical density was then read at 535 nm (Hitachi Spectophotometer). MDA was determined in mM by a standard curve created with 1, 1, 3, 3-tetramethoxy-propane (Merck). A water sample treated in the same way was applied as a blank. All determinations were performed in duplicate.

Animal sacrifice was not performed for the estimation of pro-inflammatory mediators in animals of group B. That was based on the similar survival rate (see below) of groups A and B.

### Statistical analysis

Survival of each group was estimated by Kaplan-Meier analysis; comparisons between different groups were performed by the log-rank test.

Concentrations of LPS and of serum pro-inflammatory parameters were expressed by their mean (± SE). Comparisons between groups A and C were performed by the Mann-Whitney U test. Any value of *P *equal to or below 0.05 was considered as significant.

## Results

MICs of ticarcillin/clavulanate, piperacillin, ceftazidime, imipenem, meropenem, ciprofloxacin and amikacin for the test isolate were >256/4, >256, 16, 16, 16, >256 and >512 μg/ml respectively.

**Figure 1:**
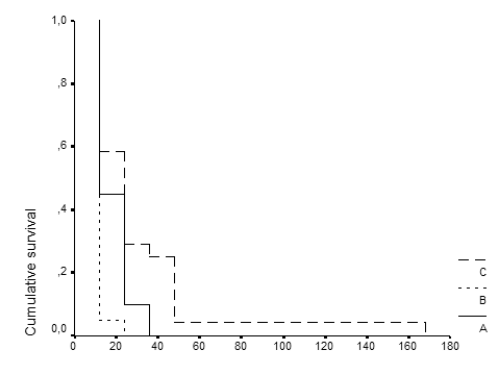
Comparative survival of Wistar rats after bacterial challenge with multidrug-resistant *Pseudomonas aeruginosa*. Group A: controls; group B: rats pre-treated with seed oil; and group C: rats pre-treated with thalidomide.

Mean (± SE) survival of group A was 18.60 ± 1.84 hours, of group B 12.60 ± 0.60 hours (*P: *0.036 compared to group A) and of group C 30.50 ± 6.62 hours (*P *equal to 0.043 when compared to group A and equal to 0.002 when compared to group B). Survival curves of each group of treatment are given in Figure 1. Survival of one animal of group C was prolonged to 168 hours. After subtraction of that animal, the mean (± SE) survival of group C was 24.52 ± 2.97 hours (*P *equal to 0.049 when compared to group A and equal to 0.004 when compared to group B).

No diarrhea was noted over follow-up of groups B and C.

All animals had positive blood cultures. Comparisons of serum pro-inflammatory mediators of groups A and C estimated five hours after bacterial challenge are given in Table 1. Mean LPS levels of groups A and C were 13.12 and 13.52 EU/ml respectively. Respective values of TNFα were 153.9 and 74.7 pg/ml, of IFNγ 782.4 and 564.4 pg/ml, of NO 1,694.4 and 618.9 μM, and of MDA 4.30 and 4.564 mM respectively.

**Table 1:** Comparison of serum concentrations of endotoxins (LPS), nitric oxide (NO), malondialdehyde (MDA), tumour necrosis factor-alpha (TNFα) and interferon-gamma (IFNγ) of animals-controls (group A) and of animals pre-treated with thalidomide (group C) sacrificed five hours after bacterial challenge.

	Group A	Group C	p
	Mean ± SE		
		
LPS (EU/ml)	13.12 ± 0.17	13.52 ± 0.44	NS
NO (μM)	1,694.4 ± 400.9	618.9 ± 350.3	0.009
MDA (mM)	4.30 ± 1.51	4.56 ± 0.54	NS
TNFα (pg/ml)	153.9 ± 53.8	74.7 ± 22.5	0.012
IFNγ (pg/ml)	782.4 ± 103.2	564.4 ± 116.5	NS

## Discussion

The perspective of an immunomodulatory intervention in sepsis, as evolved from human studies with the application of monoclonal antibodies, was very promising. However the application of these antibodies in clinical practice failed to disclose particular benefit [8,9]. The need of such immunotherapies seems to be increasing for the field of nosocomial infections where the availability of antimicrobial agents is limited. Thalidomide has been proved effective in reducing serum TNFα in experimental sepsis induced by endotoxins [10,11] and *E.coli *[5] by a mechanism differing from monoclonal antibodies. The present study documented the effect of thalidomide in an experimental model simulating a nosocomial infection ie sepsis by multidrug-resistant *P.aeruginosa*. This is the first time, to our knowledge, that an agent with anti-TNFα properties is applied in an experimental model of sepsis triggered by a multidrug-resistant pathogen.

The applied model of sepsis was lethal as documented by the absolute mortality rate of animal-controls. Results revealed a considerable benefit of thalidomide intake on survival after bacterial challenge (Figure 1). Animals administered only the vehicle of thalidomide died earlier than controls a phenomenon aggravating the potency of thalidomide. Bacteremia and endotoxemia were equally present in all groups of treatment (Table 1) so that all animals had the same chance of evolution to sepsis. Thalidomide prolonged survival even in the presence of considerable endotoxaemia.

The action of thalidomide seems to be mediated by degradation of the mRNA of TNFα and probably of IFNγ [12]. In the present study, intake of thalidomide was accompanied by significant decrease of serum TNFα and of NO whereas serum levels of MDA and of IFNγ remained unaffected (Table 1). Its specific anti-TNFα effect has also been considered as the mode of action in experimental sepsis by *E.coli *[5]. Biosynthesis of NO is triggered by TNFα [13], so that decrease of NO following that of TNFα might be expected. Thalidomide intake did not influence serum levels of MDA and of IFNγ. MDA is the end product of the peroxidation of cell membrane lipids occurring during the septic cascade; lipid peroxidation is triggered by a variety of pro-inflammatory mediators [14,15]. The specific blockade of TNFα and not of any other cytokine by thalidomide might be consistent with its failure to affect lipid peroxidation. The latter observation is also consistent with the observation that thalidomide did prolong survival but that all animals eventually died (Figure 1). However, it should be mentioned that the activity of thalidomide might be mediated by other pathways different than inhibition of TNFα.

## Conclusion

The present study revealed that intake of thalidomide considerably prolonged survival in experimental sepsis by multidrug-resistant *P.aeruginosa *an effect probably attributed to decrease of serum TNFα. These findings probably merit further research in order to elucidate their clinical relevance.

## Competing interests

The author(s) declare that they have no competing interests.

## Authors' contributions

EJGB participated in the design of the study, in the performance of estimation of inflammatory parameters and drafted the manuscript.

NB participated in the administration of the drugs and in the conduct of the animals' experiments

GL participated in the administration of the drugs and in the conduct of the animals' experiments

VP participated in the conduct of the animals' experiments

VK participated in the estimation of malondialdehyde

DP participated in study design

PEK participated in study design, in the conduct of the animals' experiments and drafted the manuscript

HG participated in study design and drafted the manuscript

## Pre-publication history

The pre-publication history for this paper can be accessed here:


